# Arthroscopic Repair Versus Conservative Treatment in Degenerative Cuff Tears: Midterm Results

**DOI:** 10.3390/life15081254

**Published:** 2025-08-07

**Authors:** Maria Rosario Camacho-Sanchez, Irene Calzado-Alvarez, Jose Carlos Minarro, Diana Maria Dussan-Arango, Clementina López-Medina, Alberto Izquierdo-Fernandez

**Affiliations:** 1 Orthopedic Surgery and Traumatology Department, Alto Guadalquivir Hospital, 23740 Jaen, Spain; 2 Faculty of Medicine, University of Córdoba, 14004 Córdoba, Spain; 3 Orthopedic Surgery and Traumatology Department, Reina Sofia University Hospital, 14004 Córdoba, Spain; 4 Rheumatology Department, Reina Sofia University Hospital, 14004 Córdoba, Spain; 5 Maimonides Institute for Research in Biomedicine of Cordoba, 14004 Córdoba, Spain

**Keywords:** arthroscopy, degenerative cuff tear, rehabilitation

## Abstract

(1) Background and aim: The benefit of surgical treatment compared to conservative management is unclear in degenerative cuff tears, and there is limited evidence regarding midterm functional outcomes. This study sought to compare the midterm functional outcomes of surgical versus conservative treatment for rotator cuff tears. (2) Methods: All patients on the waiting list for arthroscopy of cuff tears in a single center between 2013 and 2015 were analyzed. They were divided into two groups: those who underwent surgery (arthroscopy group) and those who declined the procedure (orthopedic group). The primary endpoint was shoulder functionality, evaluated with the CMS, SST, and SPADI-SP questionnaires. Inverse probability of treatment weighting (IPTW) was used to account for differences between the groups. (3) Results: Of 57 patients (67 (62–71) years old, 47% women), 32 were in the arthroscopy group and 25 in the orthopedic group. Functionality was assessed at a median of 7 (7–8) years after diagnosis. The patients in the arthroscopy group were younger (*p* = 0.023) and more frequently women (*p* = 0.074). No significant differences were observed in the type of tear (*p* = 0.205) or laterality (*p* = 0.164). Functional outcome analysis showed more favorable scores in the surgical group: constant (74.5 ± 16.6 vs. 58.4 ± 23, *p* = 0.016), SST (7.3 ± 3.1 vs. 4.9 ± 4.2, *p* = 0.016), and SPADI-SP (35.7 ± 26.6 vs. 56.1 ± 30.4, *p* = 0.006). (4) Conclusions: In this cohort of patients with cuff tears, arthroscopic repair was associated with better clinical and functional midterm results compared to conservative treatment, although the benefit was less evident in older patients and those with complete tears.

## 1. Introduction

Rotator cuff injuries are common in the general population and become more frequent with age. It is estimated that approximately 15–20% of those over 60 years of age will experience a rotator cuff tear, and there is a 50% chance of it being bilateral [1,2]. Furthermore, the prevalence of rotator cuff tears exceeds 50% among those over the age of 80. Despite this high prevalence, recent data indicate that public understanding of rotator cuff tears and their causes, treatment options, and prognosis remains limited [3].

The spectrum of injuries ranges from inflammation to rupture, which can be partial or complete. Partial cuff tears are those in which the articular side is not communicated with the bursal side; that is, the full thickness of the cuff is not involved [4]. Ellman divided them into three subtypes according to cuff thickness involvement [5]. However, this classification is rarely used in daily clinical practice, as partial tears are usually referred to as low-grade when less than 50% of the tendon thickness is affected and as high-grade when more than 50% is affected [6]. Complete cuff tears are those that affect the full thickness, connecting the articular and bursal parts [7]. Patte classified them into three stages based on cuff retraction [8].

Although some rotator cuff injuries may remain asymptomatic, they are a leading cause of shoulder pain and functional impairment, particularly among middle-aged and older adults [9]. A meta-analysis encompassing more than 3000 patients from 26 studies identified an association between symptomatic rotator cuff tears and diabetes, hypertension, higher body mass index, and smoking [10]. The long head of the bicep tendon is increasingly being recognized as a key pain generator in rotator cuff pathology. Due to its anatomical and functional connection with the rotator cuff and its stabilization by the bicep pulley, disruption of this pulley complex may lead to symptom onset and tear progression. As such, bicep tendon involvement should be considered an integral component of the degenerative process in rotator cuff disease [11,12].

The treatment of repairable cuff tears is not standardized and remains the subject of ongoing debate [13,14,15,16]. Management strategies are generally categorized as either conservative, such as physical therapy, or surgical, which may include arthroscopic repair or debridement, open rotator cuff repair, and, in selected cases, shoulder arthroplasty [17]. In PASTA (Partial Articular-sided Supraspinatus Tendon Avulsion) lesions, augmenting the supraspinatus reinsertion with the long head of the bicep tendon is a feasible option [18]. However, there are few studies comparing the results of surgical repair with those of conservative treatment. Another limitation of the existing literature is the relatively short duration of follow-up, which hinders the evaluation of sustained functional benefit over time [19,20,21,22].

Therefore, the aim of the present study was to compare the midterm functional outcomes of arthroscopic and conservative treatment in patients with cuff tears.

## 2. Materials and Methods

### 2.1. Study Design and Participants

A single-center, cross-sectional study was conducted to compare the functional outcomes of patients who underwent arthroscopic repair with those who were treated conservatively after declining the procedure. Patients were diagnosed and, if necessary, operated on between 2013 and 2015. Functionality questionnaires were administered in 2022. This study was conducted in accordance with the Declaration of Helsinki. The study protocol was reviewed and approved by the local ethics committee, and all participants provided written informed consent.

**Figure 1:**
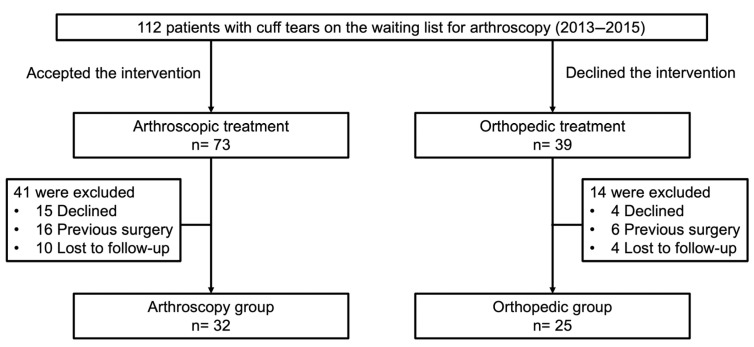
Flow diagram of the patients included in this study.

During the study period, 112 patients underwent arthroscopic cuff repair at our hospital, of whom 57 were included in this study. Reasons for exclusion are presented in the patient flow diagram (Figure 1). Of these, 32 patients underwent the procedure (arthroscopy group), and 25 who declined surgery (orthopedic group), for various reasons, were managed with conservative treatment. The inclusion criteria were (i) confirmation of partial or complete cuff rupture by ultrasound or magnetic resonance imaging, (ii) indication for arthroscopic repair, and (iii) age equal to or greater than 18 years at diagnosis. The exclusion criteria were (i) refusal to participate in this study, (ii) surgery performed at another center, and (iii) previous shoulder surgery.

### 2.2. Outcome Measures

The primary endpoint of this study was shoulder functionality, which was assessed using the Constant–Murley Score (CMS), Simple Shoulder Test (SST), and Shoulder Pain and Disability Index (SPADI-SP) questionnaires [23,24,25,26]. Secondarily, the components of the scores were analyzed separately, and pain was assessed using the visual analogue scale (VAS).

### 2.3. Clinical Assessment

A complete clinical evaluation was performed at a median follow-up of 7 years (interquartile range: 7–8 years) after diagnosis. The assessment included medical history and physical examination. The clinical examination for rotator cuff tears included inspection, palpation, range of motion testing, and specific maneuvers (e.g., the Jobe test to assess supraspinatus integrity and the belly press test for subscapularis function), as well as the Neer and Hawkins–Kennedy impingement signs to evaluate subacromial involvement. Previous clinical and surgical data were collected from the electronical medical records.

### 2.4. Surgical Procedure and Postoperative Management

Surgical treatment consisted of arthroscopic cuff repair by debridement, bone anchors, and convergence suture when necessary. In patients with tendinopathy of the long head of the bicep, tenotomy without tenodesis was also performed. Acromioplasty was performed when there was a decreased subacromial space. In cases of symptomatic acromioclavicular joint arthrosis, an arthroscopic Mumford procedure was added.

In the postoperative period, patients were immobilized in a sling without an abduction pillow. Passive exercises were started at 3 weeks and active exercises at 6 weeks. All patients were referred to a rehabilitation program. Clinical follow-up was scheduled according to the clinical course of the patient.

### 2.5. Conservative Treatment

Conservative treatment consisted of medical treatment with analgesics or non-steroidal anti-inflammatory drugs (NSAIDs), as well as subacromial infiltrations using a mixture of 4 mL of 2% mepivacaine and 40 mg of triamcinolone acetonide, combined with physiotherapy as indicated by the treating physician. A maximum of three infiltrations per year was administered. The patients were examined at regular intervals every 3–6 months. If there was no clinical improvement after 12 months, an imaging examination (magnetic resonance imaging or ultrasound) was performed to assess whether there was progression of the tear.

### 2.6. Statistical Analysis

Quantitative variables are shown as means and standard deviations or medians (interquartile range: 25th percentile–75th percentile) and were compared using the Mann–Whitney U test. Normality was assessed using the Shapiro–Wilk test and QQ plots. Qualitative variables are expressed by count and percentage and were compared by Chi square. Questionnaire scores were compared using linear regressions with inverse probability of treatment-weighting (IPTW) with weights derived from propensity scores [27]. The propensity score was calculated using a logistic regression model that was computed with the covariates that could condition differences in the functionality questionnaires based on previous studies: age, sex, type of tear (complete vs. partial), and laterality (left vs. right). Standardized mean differences before and after the weighting were used to evaluate the balance of the groups regarding the covariates. An adequate balance was considered when the standardized mean difference was <10%. To explore potential subgroup effects, we used linear regression models that included interaction terms between the treatment groups and each covariate of interest. To evaluate the robustness of the IPTW model to unmeasured confounding, we performed a sensitivity analysis using the robustness value framework. The robustness value quantifies the minimum strength of association that an unmeasured confounder would need to have with both the treatment and the outcome to substantially modify the estimated treatment effect, either by shifting the point estimate to null or by making it statistically non-significant [28]. Statistical analyses were performed using SPSS software (version 24; IBM Corp., Armonk, NY, USA) and R software (version 4.2.1; R Foundation for Statistical Computing, Vienna, Austria).

## 3. Results

### 3.1. Clinical Characteristics

In all, 57 patients were included in this study. The median age at diagnosis was 67 (62–71) years, and 27 (47.4%) were women. The arthroscopy group consisted of 32 patients and the orthopedic group of 25. The median time elapsed between diagnosis and recruitment was 95 (91–101) months. The baseline characteristics of the patients are shown in Table 1. In the arthroscopy group, the patients were significantly younger (58 (54–61) vs. 62 (55–66) years, *p* = 0.018) and more frequently women (59.4% vs. 32.4%, *p* = 0.040). There were no significant differences in laterality or in type of tear, with most tears being complete in both groups (87.5% vs. 68.0%, *p* = 0.208).

**Figure 2:**
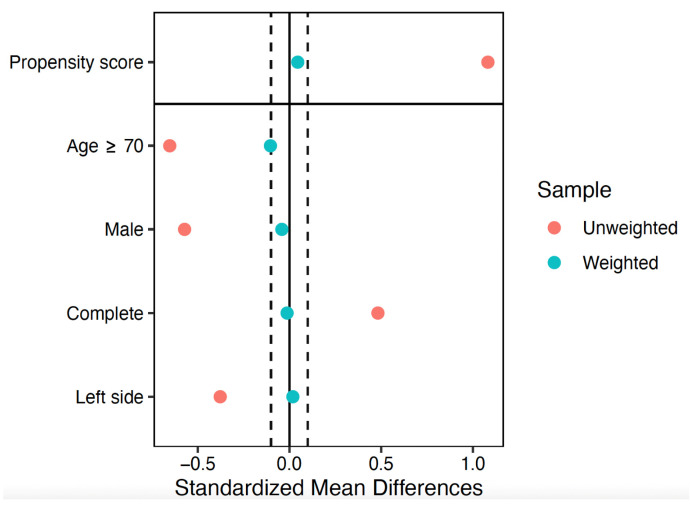
Covariate balance. Dotted lines represent ±0.1 standardized mean differences. After the weighting, a good balance was observed, with standardized mean differences between −0.1 and 0.1 for all the covariates included in the propensity score model.

The effect of the arthroscopic treatment on the functional outcomes was analyzed with regression models adjusted by IPTW. The standardized mean differences of the covariates included in the propensity score model in the unweighted and weighted samples are shown in Figure 2. Before weighting, there was a significant imbalance in most of the covariables. After weighting, the covariates showed an excellent balance, with standardized mean differences of less than 10%.

### 3.2. Functional Outcomes

**Figure 3:**
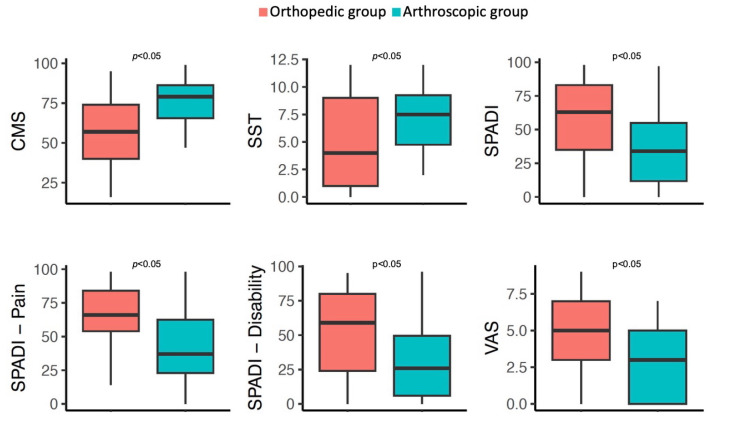
Midterm results according to treatment group: graphic representation. CMS: Constant–Murley Score; SST: Simple Shoulder Test; SPADI: Shoulder Pain and Disability Index; VAS: visual analogue scale. *p* values were obtained from IPTW—linear regression models.

The patients in the arthroscopy group had more favorable scores in all questionnaires: CMS (74.5 ± 16.6 vs. 58.4 ± 23.0, *p* = 0.016), SST (7.3 ± 3.1 vs. 4.9 ± 4.2, *p* = 0.016), SPADI (35.7 ± 26.6 vs. 56.1 ± 30.4, *p* = 0.006), SPADI—Pain (41.0 ± 28.7 vs. 63.1 ± 27.3, *p* = 0.009), SPADI—Disability (31.7 ± 26.6 vs. 51.6 ± 33.1, *p* = 0.005), and VAS (2.7 ± 2.6 vs. 4.8 ± 2.8, *p* = 0.006). The distribution of the scores by treatment group is shown in Figure 3. The IPTW models estimates are presented in Table 2. In the arthroscopy group, those who had undergone tenotomy of the long head of the bicep (25%) did not present better results in the CMS (*p* = 0.664), SST (*p* = 0.949), and SPADI-SP (*p* = 0.334) questionnaires. Acromioplasty was performed in 46.9% of the patients in the arthroscopic group and was not associated with better outcomes: CMS (*p* = 0.433), SST (*p* = 0.350), and SPADI-SP (*p* = 0.331).

**Figure 4:**
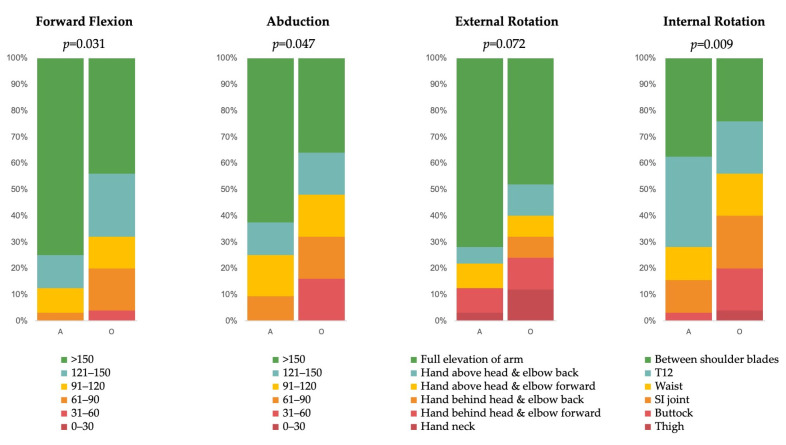
Constant–Murley Score range of motion according to treatment group. A: arthroscopy group. O: orthopedic group. T12: 12th thoracic vertebrae; SI: sacroiliac. *p* values were obtained from IPTW–linear regression models.

Patients in the arthroscopy group were more likely to have a CMS score greater than 70 points (65.6% vs. 32%, *p* = 0.036). They also had more favorable results in the individual analysis of CMS range-of-motion components: anterior flexion (*p* = 0.031), abduction (*p* = 0.047), external rotation (*p* = 0.072), and internal rotation (*p* = 0.009) (Figure 4). However, no differences in strength were observed between the two groups (6.3 ± 3.6 vs. 5.2 ± 3.6, *p* = 0.409).

On the other hand, the differences between both groups remained significant when the SPADIs for pain (SPADI-P) and disability (SPADI-D) were analyzed separately: SPADI-P (41.0 ± 28.7 vs. 63.1 ± 27.3, *p* = 0.009) and SPADI-D (31.7 ± 26.6 vs. 51.6 ± 33.1, *p* = 0.005). Regarding the pain VAS, patients who underwent arthroscopy had lower scores (2.7 ± 2.6 vs. 4.8 ± 2.8, *p* = 0.006).

Sensitivity analysis using the robustness value method showed consistently high robustness values across all outcomes (Table 3). These findings suggest that an unmeasured confounder would need to account for approximately 30% of the residual variance in both treatment assignment and the outcome to fully explain away the observed effects, or at least 8–14% to render them statistically non-significant.

### 3.3. Subgroup Analysis

**Figure 5:**
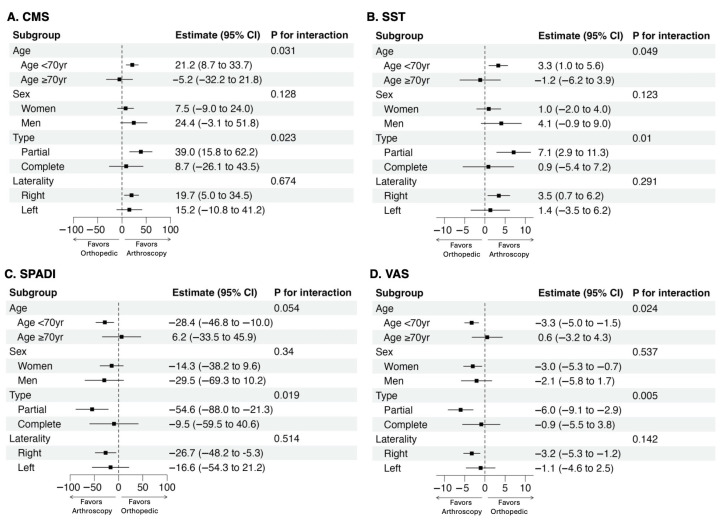
Subgroup analysis forest plot: (**A**) CMS: Constant–Murley Score; (**B**) SST: Simple Shoulder Test; (**C**) SPADI: Shoulder Pain and Disability Index; and (**D**) VAS: visual analogue scale.

The effect of arthroscopic treatment on functional outcomes was consistent across all questionnaires, as summarized in Figure 5. The positive effect of arthroscopy was observed across most subgroups analyzed. However, a significant interaction was found for age, with no apparent benefit among patients aged ≥ 70 years (*p* for interaction of < 0.05 for CMS and SST; *p* = 0.054 for SPADI). Additionally, the beneficial treatment effect of arthroscopy was attenuated in patients with complete tears, with significant interactions observed across all functional scores and the VAS for pain (*p* for interaction < 0.05).

## 4. Discussion

In this observational study of a heterogenous cohort of patients with rotator cuff injuries, arthroscopic repair was associated with better and more consistent midterm functional outcomes than conservative strategy. Functionality was objectively assessed using previously validated questionnaires. The association was consistent among the different questionnaires and was also observed after adjustment for potential confounding factors, suggesting a positive treatment effect of this procedure compared to orthopedic management. Importantly, we also identified subgroup differences in functional outcomes that may be useful for shared decision-making between surgeons and patients. In particular, the benefit of arthroscopic treatment appeared to be diminished in older patients and in those with complete rotator cuff tears.

In contemporary routine practice, conservative treatment of rotator cuff injuries is not infrequent, especially in elderly patients with degenerative or partial tears of less than 50%. Although this approach may be effective in selected patients, the indication must be carefully considered, particularly in the presence of a functionally balanced shoulder, at least in the horizontal plane, as this has been associated with better clinical outcomes [29]. Notably, conservative treatment might lead to irreversible progression of tissue degeneration so that repairable tears become irreparable [30]. As for surgical treatment, the structural failure rate is high (16–94%) despite the large number of techniques available [31,32]. Furthermore, it is not clearly known whether re-attaching the cuff to the bone prevents progression of atrophy and muscle degeneration. Previous studies support the use of surgical treatment in young patients, with complete cuff tears and partial thickness tears of greater than 50%, who are very symptomatic and have severe dysfunction [14].

In our study, we included a late-middle-age population with both partial and complete tears. After considering potential confounders, comparisons between the two strategies showed differences in the medium-term functional results in all the questionnaires that favored arthroscopic repair. A plausible explanation for this finding may be attributed to disease progression among patients in the conservative treatment group, resulting in the transformation of repairable tears into irreparable ones that are often more symptomatic [30]. This progression is frequently associated with a loss of shoulder stability or balance, particularly in the horizontal and vertical planes. Disruption of the muscular and tendinous balance around the glenohumeral joint can lead to altered biomechanics, further degeneration, and worsening symptoms, thereby reducing the potential for successful repair.

To our knowledge, three randomized clinical trials have been conducted to compare these two treatment approaches, yielding divergent results. Moreover, the heterogeneity of the study designs and the target population make direct comparisons challenging. In the trial conducted by Lambers Heerspink et al. [19], surgical repair was not associated with improved outcomes in terms of shoulder functionality assessed by the CMS at a 1-year follow-up. However, patients in the surgical group reported significantly less pain according to the VAS. In line with these results, Kukkonen et al. [20] found no positive treatment effect of surgery on functionality, as evaluated by the CMS after a two-year follow-up. In this case, no benefit was observed in terms of pain, assessed by the VAS. Accordingly, Moosmayer et al. [33] reported a neutral effect of surgery on functional results in the short and medium terms (6 months, 1 year, and 5 years). However, it is noteworthy that they observed improved shoulder functionality, evaluated by the CMS at a 10-year follow-up, in patients who underwent surgical repair. This long-term beneficial effect is consistent with our findings from patients with a median follow-up time of 8 years, further supporting the theory that conservatively treated patients may experience progression of the tear during long-term follow-up. In this regard, enlargement of complete degenerative cuff tears has been reported in 30% to 40% of cases at 3 to 4 years of follow-up [34,35]. More concerningly, postponed surgical intervention may lead to the development of massive rotator cuff tears, which are associated with higher rates of revision surgery and poorer clinical outcomes [36]. Nevertheless, it is important to consider the high failure or re-rupture rate in operated patients, which is approximately 20% after one year.

A recent study reported that shared decision-making remains limited among patients awaiting rotator cuff repair surgery [37]. Given the ongoing debate and conflicting evidence regarding the optimal management strategy, the choice between surgical and conservative treatment should be individualized and thoroughly discussed with each patient. In this context, our subgroup analyses provide potentially useful insights. Specifically, in patients aged 70 years or older, we observed no significant functional benefit from arthroscopic repair. We hypothesize that this finding may be explained by age-related changes in muscle quality. Older patients typically present with greater fatty infiltration of the rotator cuff muscles, a factor known to negatively impact the success of surgical repair and functional recovery [38,39]. However, arthroscopic treatment might be beneficial in older patients not responding to initial conservative treatment, as suggested in a recent retrospective study [40]. Additionally, the treatment effect was attenuated in patients with complete rotator cuff tears. This may be due to the increased complexity of surgical repair in the context of more extensive tendon damage, which is often associated with less favorable outcomes and a higher likelihood of postoperative re-tear [41].

Taken together, our results provide valuable insights that may assist clinicians in tailoring treatment discussions to individual patient profiles [42]. By identifying subgroups less likely to benefit from surgery, such as older adults with complete tears, these findings can help inform patient-centered decisions regarding the expected functional outcomes of arthroscopic versus conservative management over the midterm follow-up period.

Our study has some limitations that are derived from its retrospective and observational design, which limits the ability to establish causal relationships. Although we used IPTW to reduce confounding and the balance of the covariates that were included the propensity score model, was good, the presence of residual confounding due to unmeasured variables cannot be completely ruled out. Another limitation is the proportion of excluded patients in both groups, potentially introducing selection bias. Finally, no adjustment for multiple testing was performed. On the other hand, our study also has notable strengths, since it contributes to the limited body of knowledge assessing midterm functional outcomes of arthroscopic rotator cuff repair. Furthermore, we employed validated questionnaires that captured patients’ perceptions of their cuff tears and the treatment received, thus providing valuable insights into their subjective experiences.

## 5. Conclusions

Our study suggests that arthroscopic repair might offer superior midterm functional outcomes compared to conservative orthopedic treatment in patients with rotator cuff tears. Nevertheless, the observed benefit may not be uniform across all patient profiles. In particular, older adults and those with complete tears showed more limited responses to surgical intervention. These findings underscore the importance of individualized treatment strategies and shared decision-making. Further randomized controlled trials are warranted to validate these results and better define which subgroups are most likely to benefit from arthroscopic repair.

## Figures and Tables

**Table 1:** Clinical characteristics.

	Arthroscopy (*n* = 32)	Orthopedic (*n* = 25)	*p*
Age at diagnosis, years	58 (54–61)	62 (55–66)	0.018
Age at evaluation, years	65 (62–69)	69 (63–73)	0.023
Sex			0.040
Female	19 (59.4%)	8 (32.0%)	
Male	13 (40.6%)	17 (68.0%)	
Type of tear			0.205
Partial	4 (12.5%)	8 (32.0%)	
<50%	0 (0.0%)	1 (4.0%)	
≥50%	4 (12.5%)	7 (28.0%)	
Complete	28 (87.5%)	17 (68.0%)	
Grade I	6 (18.8%)	7 (28.0%)	
Grade II	13 (40.6%)	7 (28.0%)	
Grade III	9 (28.1%)	3 (12.0%)	
Laterality			0.164
Left	12 (37.5%)	14 (56.0%)	
Right	20 (62.5%)	11 (44.0%)	

Quantitative data are shown as medians (interquartile ranges) and qualitative data as *n* (%).

**Table 2:** Effect of arthroscopic treatment on functional outcomes.

	Arthroscopy (*n* = 32)	Orthopedic (*n* = 25)	Adjusted Mean Difference	Robust Standard Error	*p*
CMS	74.5 ± 16.6	58.4 ± 23.0	13.855	5.568	0.016
SST	7.3 ± 3.1	4.9 ± 4.2	2.379	0.958	0.016
SPADI	35.7 ± 26.6	56.1 ± 30.4	−20.685	7.158	0.006
SPADI—Pain	41.0 ± 28.7	63.1 ± 27.3	−19.549	7.294	0.009
SPADI—Disability	31.7 ± 26.6	51.6 ± 33.1	−22.051	7.460	0.005
VAS	2.7 ± 2.6	4.8 ± 2.8	−2.233	0.775	0.006

CMS: Constant–Murley Score; SST: Simple Shoulder Test; SPADI: Shoulder Pain and Disability Index; VAS: visual analogue scale. Group-level data are shown as means ± standard deviation. Adjusted mean differences, standard errors, and *p* values were obtained from IPTW–linear regression models. The covariables included in the propensity score model were age, sex, type of tear (complete vs. partial), and laterality (left vs. right).

**Table 3:** Sensitivity analysis for unmeasured confounding using robustness values.

	Partial R^2^	RV	RV_α = 0.05_
CMS	0.120	0.307	0.091
SST	0.110	0.295	0.076
SPADI	0.136	0.325	0.116
SPADI—Pain	0.119	0.307	0.092
SPADI—Disability	0.142	0.332	0.125
VAS	0.151	0.343	0.139

RV: robustness value. Partial R^2^ reflects the proportion of residual variance in the outcome explained by treatment after IPTW. RV indicates the minimum joint partial R^2^ an unmeasured confounder would need with both treatment and outcome to annul the observed effect, whereas RV_α = 0.05_ represents the threshold to render the effect non-significant.

## Data Availability

Data will be available upon reasonable request to the corresponding author.
